# Polyhydroxy Fullerenes (Fullerols or Fullerenols): Beneficial Effects on Growth and Lifespan in Diverse Biological Models

**DOI:** 10.1371/journal.pone.0019976

**Published:** 2011-05-27

**Authors:** Jie Gao, Yihai Wang, Kevin M. Folta, Vijay Krishna, Wei Bai, Paul Indeglia, Angelina Georgieva, Hideya Nakamura, Ben Koopman, Brij Moudgil

**Affiliations:** 1 Particle Engineering Research Center, University of Florida, Gainesville, Florida, United States of America; 2 Department of Environmental Engineering Sciences, University of Florida, Gainesville, Florida, United States of America; 3 Plant Molecular and Cellular Biology Program, Horticultural Sciences Department, University of Florida, Gainesville, Florida, United States of America; 4 Department of Materials Science and Engineering, University of Florida, Gainesville, Florida, United States of America; University of Houston, United States of America

## Abstract

Recent toxicological studies on carbon nanomaterials, including fullerenes, have led to concerns about their safety. Functionalized fullerenes, such as polyhydroxy fullerenes (PHF, fullerols, or fullerenols), have attracted particular attention due to their water solubility and toxicity. Here, we report surprisingly beneficial and/or specific effects of PHF on model organisms representing four kingdoms, including the green algae *Pseudokirchneriella subcapitata*, the plant *Arabidopsis thaliana*, the fungus *Aspergillus niger*, and the invertebrate *Ceriodaphnia dubia*. The results showed that PHF had no acute or chronic negative effects on the freshwater organisms. Conversely, PHF could surprisingly increase the algal culture density over controls at higher concentrations (i.e., 72% increase by 1 and 5 mg/L of PHF) and extend the lifespan and stimulate the reproduction of *Daphnia* (e.g. about 38% by 20 mg/L of PHF). We also show that at certain PHF concentrations fungal growth can be enhanced and *Arabidopsis thaliana* seedlings exhibit longer hypocotyls, while other complex physiological processes remain unaffected. These findings may open new research fields in the potential applications of PHF, e.g., in biofuel production and aquaculture. These results will form the basis of further research into the mechanisms of growth stimulation and life extension by PHF.

## Introduction

**Figure 1:**
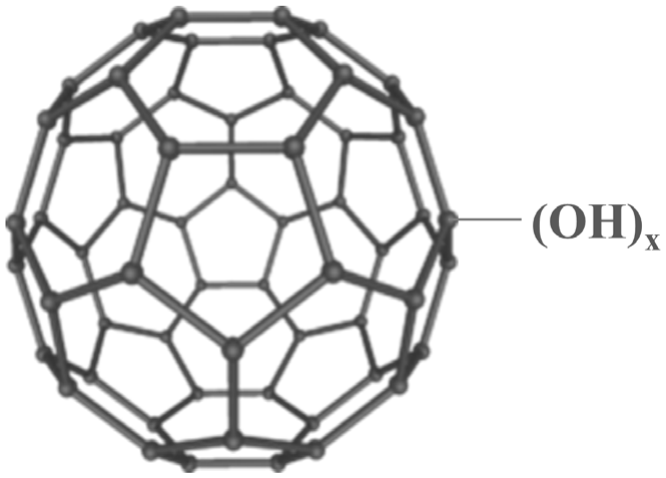
Chemical structure of a polydroxy fullerene (PHF) molecule (x = 12 to 42).

Since their discovery in 1985, C_60_ fullerenes have been among the most widely studied and used carbon-based nanomaterials due to their unique structural and electronic properties that enable numerous industrial, electrical and medical applications [1], [2], [3]. Compared with the pristine form, functionalized fullerenes have more potential applications due to their enhanced water solubility. Functionalized fullerenes have been reported to reduce oxidative stress by scavenging reactive oxygen species [4], [5] and have been examined for their antioxidant properties. Conversely, C_60_ fullerene and its water-soluble derivatives have been the subject of concern because of their rapidly growing production and potential environmental and health implications [6]. One heavily studied functionalized fullerene is the polyhydroxy fullerene (PHF, also named fullerol or fullerenol, Figure 1), wherein the fullerenes are decorated with 12–42 hydroxyl groups per molecule leading to enhanced solubility. PHF has been shown, for example, to induce the production of reactive oxygen species and cause membrane damage in rat liver microsomes [7], and exhibit cytotoxicity and phototoxicity to human epithelial cells [8], [9].

As the global production of fullerenes and their derivatives is growing rapidly, fullerene-containing products will inevitably enter various environmental theatres during their production, deployment and disposal. Among these environments, aquatic and soil systems are likely the ultimate sinks for carbon-based nanomaterials [6], [10], [11], thus exposing aquatic and soil organisms to fullerenes, especially the water soluble derivatives. Therefore, it is critical to identify the potential impacts of this nanomaterial in ecological systems.

The objective of this study was to investigate the effects of PHF on growth, development, and reproduction in representative biological systems. The study exploits the adaptive capacities, sensory spectra and/or reproductive abilities of these biological systems that ultimately serve as living sensors of bio-active compounds in their environments. Their behaviors provide valuable information that describe impacts on growth and development, and may even detail the mechanism of PHF ingress that shapes biological processes. The short-term or chronic growth and/or reproductive effects of PHF are investigated for a model freshwater phytoplanktonic organism, the green algae *Pseudokirchneriella subcapitata*, a model plant *Arabidopsis thaliana*, a model fungus *Aspergillus niger*, and a model zooplankter, the invertebrate *Ceriodaphnia dubia*.

## Results

**Figure 2:**
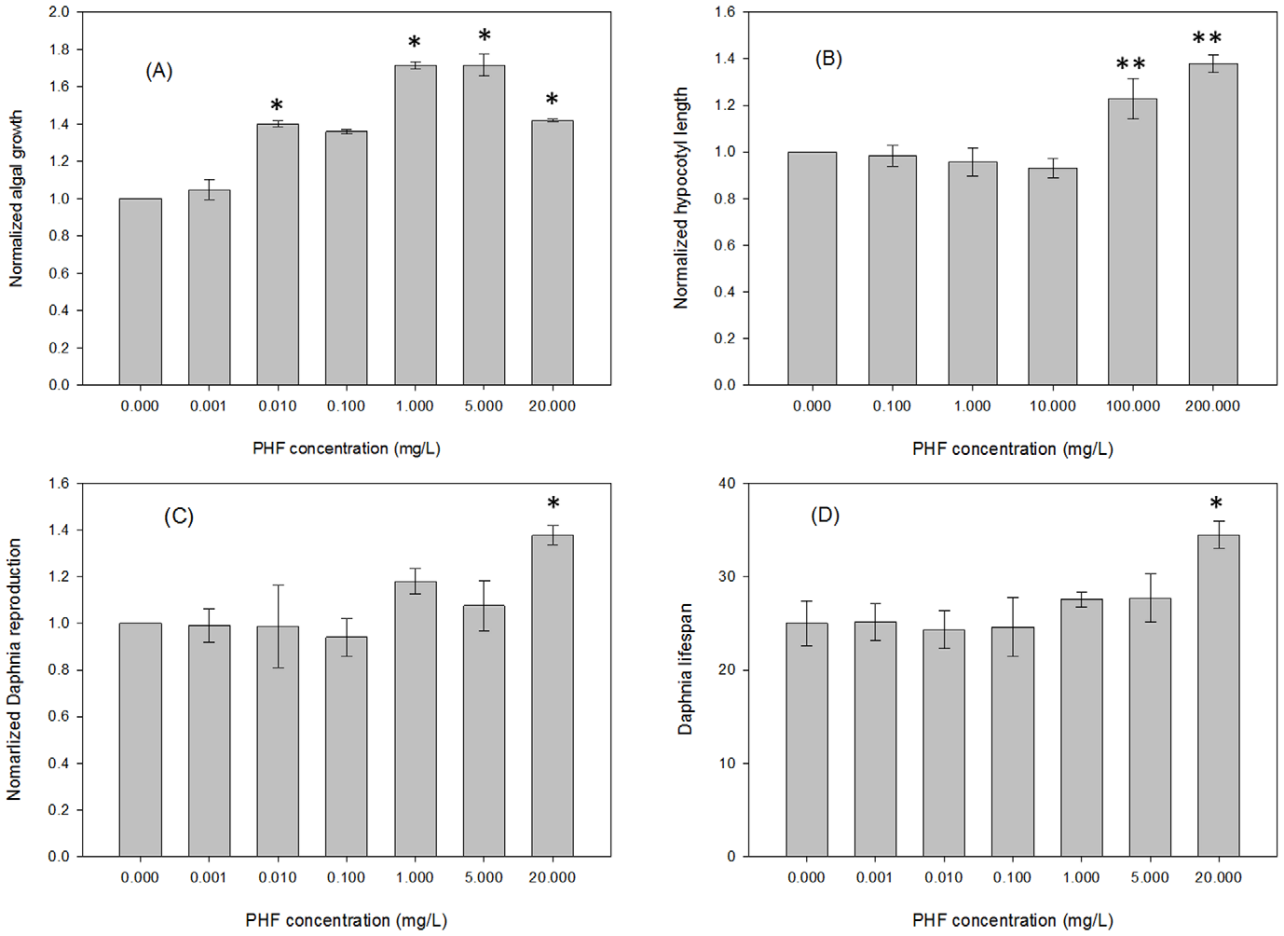
Effects of polyhydroxy fullerens (PHF) on growth and/or lifespan. (A) Normalized algal growth compared to untreated controls. The cultures were grown in the presence of the treatment for 4 days (n = 9). (B) Normalized *Arabidopsis* hypocotyl growth against untreated controls. Data represent mean of three trials, wherein each trial comprises at least 25 seedling measurements. (C) Normalized *Daphnia* reproduction as measured by the number of neonates produced over the lifetime of 5 mature daphnids (n = 4). (D) Lifespan of tested daphnids at concentrations from 0.001 to 20 mg/L (n = 20). Error bars indicate standard error of the mean. *: p<0.05; **: p<0.01.

The first tests were performed on *Pseudokirchneriella subcapitata*, a standard algal species commonly used to assess toxicity of a test substance in an aqueous system. All PHF concentrations except for 0.001 mg/L and 0.1 mg/L showed significant effects (p<0.05). After four days of growth, the number of *P. subcapitata* cells was 72% higher in the presence of 1 mg/L and 5 mg/L PHF relative to the no PHF control treatment (Fig. 2A). PHF concentrations of 0.001 mg/L and 0.1 mg/L did not have significant effects at α = 0.05, even though mean values were slightly elevated relative to controls.

**Figure 3:**
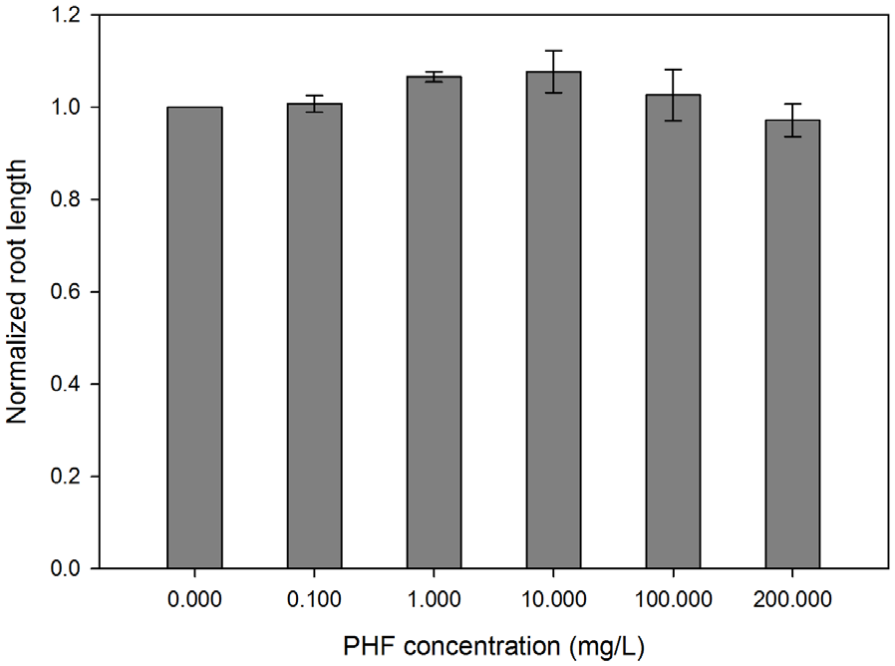
Normalized root lengths of Arabidopsis seedlings in various PHF concentrations under white light condition. No observable difference on root elongation was detected in the PHF treated seedlings compared with control seedlings (p>0.05). Root lengths were all normalized to control within each experimental replicate. At least 25 seedlings per treatment were measured in each replicate. Error bars represents the standard deviation of the three independent trials.

**Figure 4:**
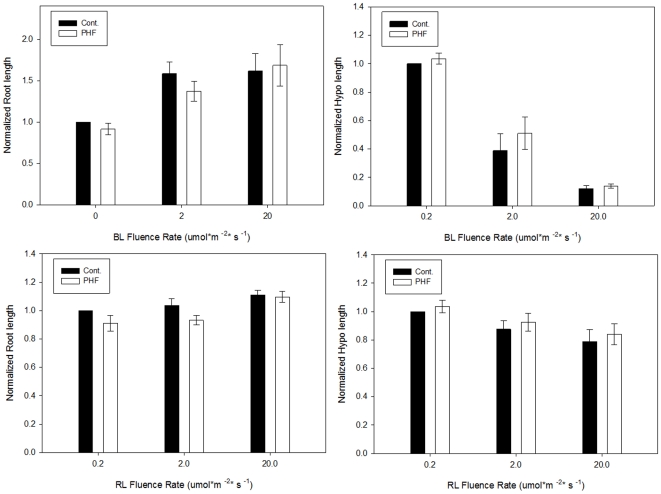
Normalized root or hypocotyl lengths of Arabidopsis seedlings under blue light (BL) or red light (RL) conditions. PHF concentration was 100 mg/L. No difference on root and hypocotyl elongation was observed under those two monochromatic light conditions (p>0.05). Root and hypocotyl lengths were all normalized to control that grown under 0.2 µmol m^−2^ s^−1^ BL or RL within each experimental replicate. At least 25 seedlings per treatment were measured in each replicate. Error bars represent the standard deviation of the three independent trials.

Light has a profound effect on the regulation of plant growth. The effects are most salient in the developing seedling. Discrete portions of the electromagnetic spectrum, nutrients, toxins and other environmental factors oftentimes have potent effects on hypocotyl (stem) growth. The developing seedling is therefore an excellent biosensor to describe how a given compound may contribute to growth responses. Due to its short life cycle, well defined physiology and substantial genetic resources, *Arabidopsis thaliana* is one of the popular plant models for plant biology and genetic research. In this study, the root and hypocotyl lengths of Arabidopsis seedlings were measured after four days of growth on various PHF concentrations. The results indicate that seedlings germinated in the presence of 100 and 200 mg/L PHF have significantly (p<0.01) longer hypocotyls compared to lower concentrations or controls (Fig. 2B). No effects were observed in root elongation (Fig. 3) or on phototropic growth (not shown). The difference in hypocotyl elongation was not observed in darkness or monochromatic light conditions (Fig. 4). The results indicate that the effects on seedling growth are specific, depend on integration of signals multiple photosensory pathways, and affect only discrete plant organs.

**Figure 5:**
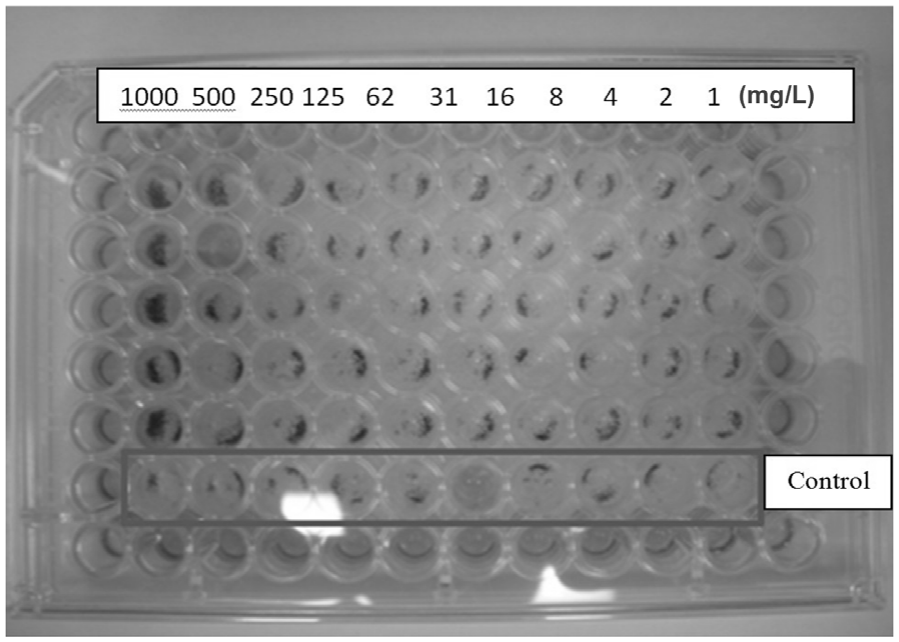
A photographic reproduction of a 96-well plate test after 120-hour incubation. The column of test cells having *A. niger* exposed to 1,000 mg/L PHF shows significantly higher growth than control or other PHF quantities.

The effects of PHF were assessed on the *Aspergillus niger*, the causative organism of black mold. We observed that concentrations above 10 mg/L stimulated growth. Treatment of *A. niger* with PHF for 120 hours (media: amended RPMI 1640) caused more spores to form compared to controls (Fig. 5), indicating that PHF at higher concentration can stimulate reproduction.

**Figure 6:**
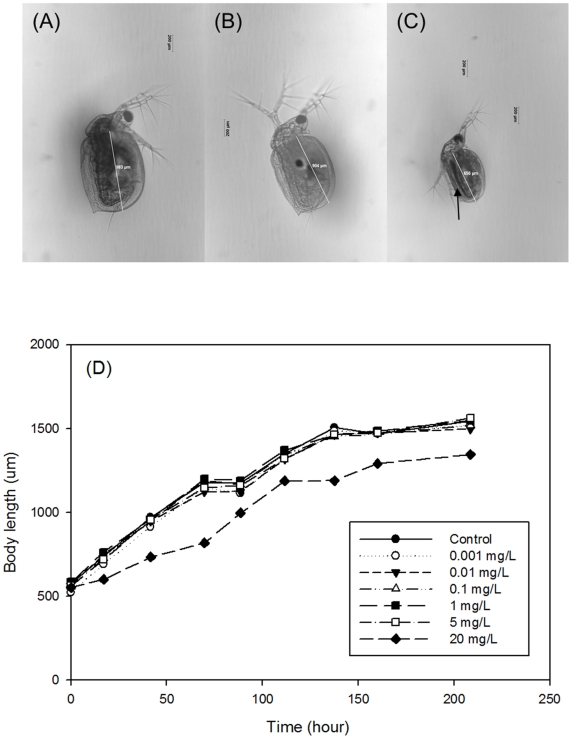
Microscopic images (10X magnification) of daphnids exposed to control and pH treatments after 2 days. (A) Control, (B) treatment with 0.001 mg/L of PHF, and (C) treatment with 20 mg/L of PHF. (D) Mean body length of daphnids over a duration of 8 days in response to various concentrations of PHF (n = 2).

The growth and reproductive effects of PHF were also tested on *Ceriodaphnia dubia*, an established EPA- recommended freshwater biological invertebrate system. PHF treatment over the lifetime of the daphnids increased the number of neonates at 20 mg/L PHF by 38% (Figure 2C). The daphnids were monitored for PHF effect on lifespan. At 20 mg/L of PHF their lifespan increased 38% over comparable non-PHF treated controls (p<0.05) (Figure 2D). Lower PHF concentrations had no significant effect. Figures 6a-c show sample micrographs taken for the daphnids in the control, 0.001 mg/L and 20 mg/L of PHF treatments after 2-day exposure. Accumulation of PHF was clearly observed in the *Daphnia* GI tract exposed to 20 mg/L of PHF (Figure 6c; arrow). During the first 8 days, the body length of each tested daphnid was measured daily and the length was determined from the base of the tail spine to the base of the head [12] (white lines in Figure 6a–c). As shown in Figure 6d, there were no observable effects on the *Daphnia* growth at concentrations of 5 mg/L or lower. At 20 mg/L of PHF, the growth rate was affected within the first hours of treatment, but was mostly unaffected thereafter, resulting in a smaller daphnid size over the test period of 8 days (α = 0.05).

## Discussion

Although it is unclear how PHFs affect cellular growth or expand lifespan, the unexpected observations may be attributed to the fact that PHF is one of the most potent ROS scavengers and could reduce oxidative challenge experienced by organisms during aging and growth [13], [14]. It was found that fullerene cage can absorb up to 6 electrons and disperse them through the 20 benzene rings over its surface [15]. In a study by Quick et al. [16], another type of functionalized fullerene, carboxyfullerene, which is a known antioxidant, has been shown to extend the lifespan of mice by 5%, and to reverse age-related cognitive impairment. However, no clear correlation has been observed between antioxidants and lifespan in the literature, so far, and other mechanisms may be responsible for the lifespan effect [17].

Recent discoveries that certain species of fungi can decompose PHF and incorporate them into biomass [18] and that PHF can be mineralized to other inorganic carbon by direct photolysis [19] shed light on other possibilities that algal, fungal or plant cells may be able to utilize PHF molecules as a nutrition source. In addition, the fact that stem growth is affected under light conditions, but root growth, phototropic curvature and dark stem growth are not affected (Figures 2B, 3 and 4), indicates that PHF effects are mechanistically discrete and not simply pleiotropic effects of a generally toxic compound. These results indicate that PHF effects are specific and are affecting seedling growth due to interaction with growth regulatory processes, particularly those associated with stem elongation in light. The principle effect that is seen is a longer stem in PHF treated seedlings. The effect is not light-quality specific, so the effect is likely in response to light, rather than sensing of light. Examples of such response pathways may include the gibberellic acid sensing/response mechanism, a cell elongation system that is inhibited by light. PHF makes hypocotyls longer, suggesting that the compound is possibly interacting with the mechanisms that adjust stem growth in the light environment, such as a hypersensitivity to, or production of, plant hormones.

The slight reduction of *Daphnia* body length in the 20 mg/L PHF (Fig. 6) treatment indicates a possible delay in aging caused directly [16] or indirectly (e.g., dietary restriction at this concentration, which has been known to extend lifespan in a variety of organisms [20], [21], [22] by a high dose of PHF. In addition, the increase and improvement of algal biomass may add favorable nutrients to the *Daphnia* food and thus increase their lifespan.

From bacteria to mammalian cells to mammalian systems, polyhydroxy fullerenes have generally been described as toxic [9], [23], [24], [25]. The results obtained from this study are unexpected because PHF functioned often as a growth stimulant, or at least a growth regulator. Our findings also suggest that application of PHF has discrete biological consequences that are species and tissue specific. Thus, it is important to note that PHF's effects can be generally regarded as beneficial, with no evidence of deleterious effects described for other nanomaterials. These findings are significant because they indicate that PHF has the potential to serve as a growth regulator or modulator of biological processes that span several kingdoms, opening the door to new applications that may benefit industry and agriculture. Such applications may include instances where growth stimulation is desired, for instance, in algal production for biofuel application, aquaculture, or in modulating plant stature or development.

## Materials and Methods

### 1. Preparation of PHF solution

PHF used in the algae and *Daphnia* study were purchased from Nano-C, Inc. (Westwood, MA), and from BuckyUSA (Houston, TX) for the fungal study, both samples with assigned possible composition C_60_(OH)_24–26_. PHF were also synthesized through an alkali route [26] and used in the plant study. Briefly, in a dry 250 ml round bottom flask 160 mg C_60_ was mixed with 55 ml toluene (Aldrich) and stirred for 30 min covered with rubber septum and equipped with Nitrogen filled balloon. To this dark purple solution was added 0.3 ml 40% aqueous tetrabutyl ammonium hydroxide (Fisher Sci) followed by addition of 5 ml 50% aqueous NaOH (Acros). Reaction mixture was stirred at room temperature for 5 days until the purple color disappeared. Then flask was placed in a freezer (−10°C) and the clear toluene layer was decanted out of the frozen mixture. To the mixture was added 40 ml nanopure water and mixture stirred at room temperature for 2 days. The dark brown mixture was freeze-dried to remove the water and the remaining powder was washed with methanol 5X20 ml. It was centrifuged and methanol was decanted each time. The residual powder dried in vacuum oven at room temperature (340 mg) and purified on neutral Sephadex size exclusion column prepared with pure water and 20 g Sephadex G-25 (Sigma-Aldrich). Collected were 25 ml fractions, dark fractions 1–3 were combined and freeze-dried to give 200 mg dark brown powder, completely soluble in water. Prior to use in this study, the PHF solutions were characterized as described elsewhere [27].

A stock solution containing 1000 mg/L of PHF was prepared by dissolving 10 mg of PHF in 10 mL of Nanopure® water and the resultant solution displayed a characteristic dark brown hue.

### 2. 96-hour Pseudokirchneriella subcapitata Growth Assay

Maintenance of the algal culture was described in Griffitt et al. [28]. Briefly, the culture medium was prepared from stock solutions according to EPA standard method for preliminary algal assay procedure [29]. The pH of the culture medium was adjusted to 7.5±0.1 with 0.1N NaOH or 0.1N HCl and then filtered through a 0.45 µm membrane and sterilized by autoclaving. A pure culture of *P. subcapitata* was obtained from Hydrosphere Research (Alachua, FL) and grown in Preliminary Algal Assay Procedure (PAAP) medium with EDTA at 25±1°C. Light source (86±8.6 µE m^−2^s^−1^) and continuous aeration were provided 24 hours per day. New cultures were prepared every week under sterile conditions by transferring approximately 20–30 mL of the mature cultures to 1–2 L of fresh sterile media.

The growth assay was performed in autoclaved 125 mL Erlenmeyer flasks according to the EPA protocol [29]. All sample dilutions (i.e. culture media spiked with increasing concentrations of PHF from 0.001 to 20 mg/L) and negative controls were run in triplicates and inoculated with 1 mL of a 4 to 7-day old algal cultures. All flasks were placed under the fluorescent lights in the same growth conditions. The algal growth after 96-h was determined by cell number count between 3.4 and 8 µm [30] using a Coulter Multisizer III (Beckman Coulter, Inc. Brea, CA, USA).

### 3. *Arabidopsis* Growth Experiments

*Arabdopsis thaliana* seeds (ecotype Columbia-0) were placed onto a minimal medium (1 mM KCl, 1 mM CaCl_2_; solidified with 1% phytoagar) containing 0–200 mg/L PHF in square Petri dishes and stratified at 4°C for 48 h. The seeds were then treated with white light (20 µmol m^−2^ s^−1^) for 15 minutes to synchronize germination. Then the plates were moved to experimental conditions in a vertical position such that seedlings would grow upright on the agar surface. The experimental conditions were darkness, white light (cool white fluorescent; 20 µmol m^−2^ s^−1^), blue or red LED light (various fluence rates tested for blue and red light at 0.2, 2 or 20 µmol m^−2^ s^−1^). Seedlings were grown for 96 h in experimental conditions, imaged on a flatbed scanner, and then measured using Image Tool 3.0 against known standards. At least 25 seedlings were measured per treatment in at least three independent experimental replicates.

### 4. Fungi Growth Experiments

Fungi growth experiments were conducted in a 96-well plate by following standard micro-dilution protocol with two different growth media: RPMI 1640 with 2% glucose and RPMI 1640 with potato dextrose broth. The protocol involves preparing a series of PHF dilutions in a 96 well plate, inoculating the wells with *A. niger*, and using the absorbance at 600 nm as an indicator of a biomass concentration. Visible light absorbance correlates to biomass to indicate fungal growth or inhibition relative to the absorbance measured from a control without PHF as taught in Schwalbe et al. [31].

Pre-prepared RPMI-1640 was obtained from Mediatech Inc. (Manassas, VA). Potato dextrose broth was prepared in the laboratory. PHF stock solutions were prepared in the culture media at concentration of 2000 mg/L, followed by 10 fold dilution with the respective media to give concentrations of 200, 20 and 2 mg/L. A serial dilution of the PHF stock solution was carried out in 96 well plates. Each plate comprised 12 columns and 8 rows. Each cell was identified by its row letter (A–H) and column number (1–12). Thus, the top, left most well would be cell A1, and the bottom, right most cell would be cell H12. After dilution, inoculation of *A. niger* was carried out following the standard protocol described in Schwalbe et al. [31]. The plates were covered with aluminum foil, kept in an orbital shaker with temperature at 37°C and 75 RPM.

### 5. Survival and Reproduction Assay Using *Ceriodaphnia dubia*

Maintenance of the *Daphnia* culture was described in Gao et al. [10] and Griffitt et al. [28]. Briefly, moderately hard water (MHW) prepared following the EPA standard method [29] was used as culture media in this test. Pure culture of *C. dubia* was obtained from Hydrosphere Research (Alachua, FL) and kept in 1 L beakers containing 500 mL of MHW in a Pervical™ model # E-30 BX environmental chamber at 25°C with constant aeration. The photoperiod was 16-hr light/8-hr dark. *C. dubia* was fed with concentrated *P. subcapitata* cells and YCT (made from yeast, cereal leaves and trout chow). The daphnids were fed every other day with 6.67 mL YCT and 6.67 mL algae solution/L culture. Neonates of less than 24 hours were separated from adults daily and used for testing.

The survival and reproduction assays were performed according to EPA's protocol [29]. MHW served as negative control and as the diluent to prepare media with increasing concentrations of PHF. Neonates less than 24 hours were separated from adults and fed 2 hours prior to test start. For each test, groups of 5 *Daphnia* neonates were transferred into 30 ml plastic cups containing 20 ml of MHW (controls) or MHW plus PHF at various concentrations (i.e. 0.0001, 0.001, 0.01, 0.1, 1, 5 and 20 mg/L) for 7 days to assess the effects of PHF on growth, survival and reproduction. The daphnids were cultured at 25±1°C with the same photoperiod and fed with 40 µL of YCT and *P. subcapitata* daily per daphnid. The culture medium was renewed three times a week. Every day the survival and number of newborns were recorded and the offsprings produced were separated from the test containers. All sample dilutions and negative controls were run in four replicates and each treatment had a total of 20 daphnids.

### 6. Growth effects of PHF on *Ceriodaphnia dubia*

At each concentration, two neonates were kept in separate cups (i.e., one per cup) at same conditions as other test daphnids and observed by optical microscopy 1 hour after feeding every day. The micrographs taken were analyzed for size (i.e. core body length) of exposed daphnids and distribution of PHF in the body.

### 7. Effect of PHF on the lifespan of *Ceriodaphnia dubia*

The same daphnids used in the survival and reproduction assay were kept and tested for their lifespan with and without the exposure to PHF. The daphnids were cultured and fed in the same manner as previously described. The culture medium was renewed three times a week. Every day the survival and number of newborns were recorded and the offsprings produced were separated daily until the death of all daphnids.

### 8. Data Analysis

All the experiments were run in triplicate or greater, and data were illustrated as mean ± standard error (SE). Statistical analyses were performed using one-way analysis of variance (ANOVA) followed by Dunnett's test with an NCSS 2004 (Kaysville, Utah). A *p* value of less than 0.05 was considered to be statistically significant.
